# Adapting and validating the FOODLIT-Tool in Turkish: a psychometric study on food literacy for sustainability

**DOI:** 10.1017/S1368980026102821

**Published:** 2026-06-03

**Authors:** Aslıhan Atar, İrem Nur Şahin Anılgan, Halime Pulat Demir

**Affiliations:** 1 Nutrition and Dietetics, Istanbul Beykent Universitesi, Türkiye; 2 Istanbul Medipol University - Kavacık Campus, Türkiye; 3 Gazi University, Türkiye; 4 Istanbul Beykent University - Beylikduzu Campus, Türkiye

**Keywords:** Food literacy, sustainability, psychometric validation, nutrition, Turkish adults

## Abstract

**Objective:**

This study aimed to adapt and validate the FOODLIT-Tool in Turkish to assess sustainable food literacy among Turkish adults. The adaptation addresses a lack of psychometrically validated instruments for measuring food literacy and sustainability-oriented eating behaviours in Turkey.

**Design:**

A psychometric evaluation was conducted, including forward-translation, back-translation and cultural adaptation steps, followed by expert review for content validation. Construct validity was assessed using confirmatory factor analysis (CFA), and internal consistency was evaluated to ensure reliability.

**Setting:**

Data collection was conducted online via Google Forms between July and August 2024.

**Participants:**

A total of 482 participants aged 18 to 65 years residing in Turkey voluntarily completed the Turkish version of the FOODLIT-Tool, along with a general information form and the Sustainable and Healthy Eating Behavior Scale.

**Results:**

CFA results demonstrated an acceptable model fit (*χ* ^2^/df = 3·67, CFI = 0·91, RMSEA = 0·07). Cronbach’s α values ranged from 0·60 to 0·91 across the sub-dimensions, indicating adequate to excellent internal consistency. Significant positive correlations were found between FOODLIT subscales and sustainable eating behaviours. Women scored significantly higher in culinary competence, production quality and environmental awareness.

**Conclusions:**

The adapted Turkish version of the FOODLIT-Tool demonstrates satisfactory validity and reliability for evaluating food literacy and sustainable dietary practices among Turkish adults. The scale serves as a useful assessment tool for future research and intervention studies focusing on sustainable nutrition. Further validation using representative samples and longitudinal designs is warranted before its application in public health or policy-making contexts.

## Introduction

Sustainable nutrition is a dietary pattern that promotes health for current and future generations while contributing to food security and having low environmental impacts. According to the FAO, sustainable diets protect biodiversity and ecosystems, are culturally acceptable, accessible, economically fair, affordable, nutritionally adequate, safe and healthy while optimising natural and human resources^(1,2)^. Sustainable nutrition aims to minimise the ecological footprint associated with food production and consumption while also promoting the intake of locally sourced, nutrient-dense foods^(3)^.

In Turkey, increasing urbanisation, changes in dietary patterns and a growing reliance on imported food products have contributed to environmental challenges such as food waste and greenhouse gas emissions. Additionally, awareness of sustainable nutrition remains relatively low among the general population, with many individuals prioritising convenience and cost over ecological impact. Educational initiatives targeting this gap are limited, underlining the need for effective tools to assess and enhance sustainable nutrition awareness at a societal level.

Promoting sustainable nutrition awareness through education facilitates individuals and communities in making informed decisions on this issue. Educational programmes that enhance food literacy have been shown to help individuals understand the environmental and health impacts of their food choices^(4)^. Sustainable nutrition literacy is essential in shaping conscious dietary choices and promoting environmentally sensitive eating behaviours^(5)^. Environmentally conscious eating behaviours encompass the essential knowledge and skills that enable individuals to improve their health and make decisions supporting the sustainability of the planet and food systems^(4,6)^.

Recent studies show that individuals with high levels of nutrition literacy are more likely to adopt sustainable eating behaviours, which have the potential to both enhance diet quality and reduce environmental impacts^(4,7,8)^. Despite global efforts to promote sustainable diets, there is a lack of localised, culturally relevant tools to measure and enhance sustainable nutrition literacy. Addressing this gap is critical for fostering informed dietary choices that support both personal health and environmental sustainability. The FOODLIT-Tool is a comprehensive scale developed to measure food literacy, which encompasses an individual’s knowledge, skills and attitudes related to food choices, health and sustainability^(5)^. It has been widely recognised for its ability to assess multiple dimensions of food literacy, including ecological and ethical considerations. Although scales adapted previously exist to assess sustainable nutrition literacy in Turkey^(9)^, this study has conducted extensive psychometric analyses on adapting the FOODLIT-Tool into Turkish. The adaptation of the FOODLIT-Tool to Turkish not only provides a reliable instrument for assessment but also supports broader initiatives aimed at improving sustainable eating habits in Turkey. This study, therefore, contributes to both academic literature and public health efforts by enabling a comprehensive understanding of sustainable nutrition in a culturally relevant manner.

The purpose of this study is to evaluate sustainable eating behaviours in Turkish society through a multidimensional approach that includes knowledge level, application and ethical awareness. This study hypothesises that individuals with higher levels of sustainable nutrition literacy, as measured by the adapted FOODLIT-Tool, are more likely to exhibit sustainable eating behaviours. Furthermore, it is expected that the psychometric adaptation of the FOODLIT-Tool will demonstrate reliability and validity in the Turkish context, enabling accurate measurement of sustainable nutrition literacy and its relationship with dietary behaviours. Adapting the FOODLIT-Tool to Turkish offers the potential to measure individuals’ sustainable nutrition awareness comprehensively, supporting sustainable eating habits at both individual and societal levels.

## Materials and methods

### Participants

The study population consists of adult individuals aged 18–65 years. The literature recommends that the sample size for validity and reliability studies be five to ten times the number of scale items^(10)^. Accordingly, for this study, which uses a twenty-four-item scale, a minimum of 120 to 240 participants was targeted. However, the final sample size of the study was increased to 482 participants, exceeding the initially recommended size. This adjustment was made to ensure broader representation and improve the reliability of the findings. The observed trends and relationships remained consistent, indicating that the increased sample size strengthened the validity and robustness of the study’s conclusions.

A survey prepared via Google Forms was used as the data collection tool. The survey was distributed to participants via email and WhatsApp, and participants were selected using the snowball sampling method. Individuals who agreed to complete the survey first approved an informed consent form and then completed the survey online. The criteria for participation included being 18 years or older, residing in Turkey, having a sufficient command of the Turkish language to understand and complete the survey, having no physical or mental health conditions that could prevent survey completion and voluntarily agreeing to participate in the study by providing informed consent. These criteria were established to ensure that the sample included individuals who could reliably understand and respond to the survey questions and to maintain consistency in the study population.

### Study design

**Figure 1.**
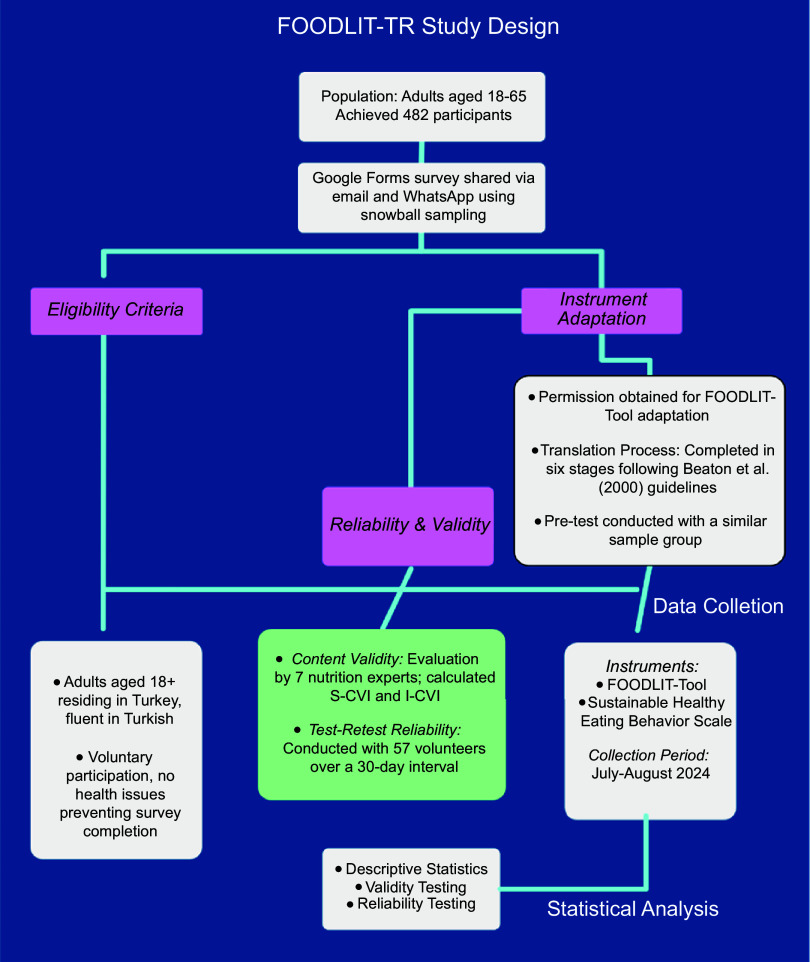
Figure 1 long description. Study design.

For this study, permission was obtained via email from Rosas, the original developer of the FOODLIT-Tool, to evaluate its Turkish psychometric properties^(11)^. Initially, the scale was adapted to Turkish and underwent a cultural adaptation process. In the cross-cultural adaptation of the scale, guidelines recommended by Beaton and colleagues were followed^(12)^. Accordingly, the translation process was completed in six stages: translation, synthesis, back-translation, expert committee review, pre-test and final version. After achieving language validity, a pre-test was administered to a sample group with similar characteristics. The final survey form, which received no negative feedback, was presented to participants after the pre-test. The test-retest method was applied to fifty-seven participants for reliability analysis to evaluate the scale’s reliability. The steps involved in the scale adaptation process are presented in Fig.1.

Data collection was conducted between July and August 2024. The survey form administered to participants included demographic information (age, sex, education level and health status), the FOODLIT-Tool and the Sustainable and Healthy Eating Behavior Scale. Ethical approval for the study was obtained from the Ethics Committee for Non-Interventional Research at İstanbul Beykent University, with approval number E-10840098-202.3.02-3727, dated 25 June 2024. This research was carried out by the principles of the Declaration of Helsinki and the rules of ‘research and publication ethics.’

#### Scale language validity

Since the original scale was in English, three researchers translated it into Turkish. Following the initial translation, four faculty members from İstanbul Beykent University’s Faculty of Health Sciences reviewed the translated text. Different translation versions were compared at this stage, and adjustments were made in terms of meaning, expressions, concepts, language and context to create a single Turkish version.

This draft form was then subjected to detailed examination by three faculty members from the Department of English Translation and Interpreting at İstanbul Beykent University. Subsequently, the back-translation method was applied by a lecturer who teaches health sciences courses in English, translating the Turkish version back into English. The items in the back-translated form were compared with the original scale; any unsuitable items were revised with the input of researchers, advisors and two faculty members with advanced English proficiency. The final version of the form was then submitted for expert review.

#### Content validity

In the literature, it is recommended to gather feedback from at least three to twenty experts for content validity assessment^(13)^. In this study, the draft form created after the translation procedures was presented to seven faculty members specialising in nutrition and dietetics to evaluate the scale’s content validity. The content validity index (CVI) and item content validity index (I-CVI) were calculated using the method suggested by Polit and Beck^(14)^. Experts were asked to evaluate both the Turkish-translated scale and the original version.

For each item, the CVI was used in the analysis of expert opinions, with items rated on a scale of 1 to 4 as follows: 1 = ‘not suitable,’ 2 = ‘somewhat suitable, requires modification,’ 3 = ‘quite suitable, minor changes needed’ and 4 = ‘very suitable’^(13)^. Necessary adjustments were made to the scale items based on expert feedback, and each item was finalised. Experts evaluated each item as ‘suitable,’ ‘can remain’ or ‘not suitable.’ The S-CVI and I-CVI were calculated separately for each item, with scores of 1 and 2 revised per expert recommendations^(13)^. The I-CVI ranged between 0·89 and 0·99, while the S-CVI was 0·94, indicating consistency.

#### Pre-test

The literature on validity and reliability studies recommends that the scale be administered to twenty to thirty individuals with similar characteristics who are not part of the primary study sample^(15)^. Accordingly, after ensuring content validity, the original English version and the Turkish-translated form were sequentially administered to twenty-four participants in Turkey who had achieved passing scores in national language proficiency exams. These participants, aged between 20 and 50 years, were Turkish nationals residing in urban areas and represented various educational and professional backgrounds, ensuring a demographically and culturally diverse group. Data from this administration were not included in the main study.

Following the pilot application, participants’ understanding of the scale items was assessed, and no negative feedback was received for any item. After this process was completed, validity and reliability analyses of the scale commenced.

#### Test-retest reliability

After ensuring the scale’s language validity and finalising its form, its reliability was assessed through test-retest and internal consistency analyses. To examine the test-retest reliability, the final Turkish version of the FOODLIT was administered twice, 30 d apart, to fifty-seven adult volunteers. This method tested the scale’s consistency and reliability over time.

### Data collection tools

#### FOODLIT-Tool

The FOODLIT-Tool is an adaptable food literacy instrument to promote sustainability in global food systems [11]. This scale was developed to measure food literacy, its determinants and influencing factors in adults. Its significance lies in providing an innovative, multidisciplinary tool to assess adults’ food literacy and encourage behaviour changes related to food^(16)^. The scale promotes more informed dietary choices among individuals and communities by evaluating the determinants and influencing factors of food literacy. The scale comprises twenty-four items across five sub-dimensions: Culinary Competence, Choice and Planning, Production and Quality, Environmental Responsibility and Origin.

All items in the scale are structured with a four-point Likert scale reflecting frequency (0 – never; 1 – sometimes; 2 – often; 3 – always) or agreement (0 – strongly disagree; 1 – disagree; 2 – agree; 3 – strongly agree). Each item is scored between 0 and 3 points. Total and sub-dimensions scores are obtained by summing the relevant items^(11)^.

#### Sustainable and healthy eating behaviour scale

Sustainable nutrition encompasses reducing food waste, preferring local and seasonal products, plant-based diets and consuming minimally processed foods. The Sustainable and Healthy Eating Behavior Scale was developed in 2019 by Żakowska-Biemans et al. to assess individuals’ sustainable and healthy eating habits^(17)^. The scale offers a comprehensive evaluation by addressing eating habits’ sustainability and health aspects. Its Turkish validity and reliability study was conducted by Köksal et al. in 2023^(18)^. The scale comprises thirty-two items across seven sub-dimensions: Quality Marks (Local and Organic), Seasonal Foods and Avoidance of Food Waste, Animal Health, Reduction of Meat Consumption, Healthy and Balanced Eating, Local Food, and Low Fat.

Factor scores are calculated by averaging the scores for items within each factor (ranging from one to seven points). The overall scale score is obtained by averaging the scores across all factors^(17)^.

### Statistical analysis

Descriptive statistics for categorical variables are presented as frequencies and percentages. The normal distribution of numerical variables was assessed with the Shapiro–Wilk test. Summary statistics for numerical variables are presented as mean ± sd (X̄ ± s d) and minimum and maximum values. The correlation between the original and Turkish-translated scales was examined as a preliminary assessment of language validity using Pearson’s correlation coefficient. Exploratory factor analysis (EFA) was conducted to assess the construct validity of the data. The Kaiser–Meyer–Olkin and Bartlett’s tests were used to evaluate the suitability of the data for factor analysis. A Kaiser–Meyer–Olkin value of 0·70 and above indicates suitability for EFA^(19)^, while a value higher than 0·60 is the minimum threshold for factorisability. Additionally, a significant Bartlett’s test result confirms that the data are appropriate for factor analysis^(15)^. Exploratory and confirmatory factor analyses were conducted sequentially to examine the construct validity of the scale. EFA was first applied to identify the underlying factor structure of the Turkish version of the scale. Subsequently, confirmatory factor analysis (CFA) was performed on the same dataset to evaluate the theoretical plausibility and model fit of the factor structure identified through EFA. Due to sample size considerations, independent subsamples could not be created for cross-validation purposes; therefore, the CFA results should be interpreted as preliminary evidence supporting the proposed factor structure^(20)^.

Confirmatory factor analysis was performed to evaluate the model’s fit to the data and goodness-of-fit indices, including *χ* ^2^/df, Comparative Fit Index (CFI), Goodness of Fit Index (GFI) and Root Mean Square Error of Approximation (RMSEA), were examined. The internal consistency and reliability of the scale were evaluated using Cronbach’s α and Spearman–Brown coefficients. Cronbach’s α measures how consistently a scale performs as a single measurement tool without requiring multiple applications^(19)^. It is frequently used in scale development and adaptation studies to determine internal consistency ^(15)^. Finally, correlation analyses were conducted using Pearson’s correlation coefficient to examine relationships between subscales and sustainable eating behaviours.

Although split-sample cross-validation is often recommended in scale development research, it is not considered a strict methodological requirement, particularly in theory-driven validation studies^(21)^. Cross-validation is especially critical when a factor structure is generated solely through exploratory procedures; however, when confirmatory analysis is conducted to evaluate an already established theoretical model, a single adequately powered sample may be acceptable^(22)^.

In the present study, the FOODLIT-Tool has a previously validated and theoretically grounded multidimensional structure. Therefore, the CFA was conducted to assess the plausibility and structural stability of this predefined model within the Turkish context rather than to confirm a newly derived exploratory solution^(23)^. Although the total sample size (*N* 482) was sufficient for factor analytic procedures, dividing the dataset into independent subsamples would have reduced statistical power and may have resulted in less stable parameter estimates within each subgroup^(21)^.

## Results

Table 1 presents the socio-demographic characteristics of the 482 participants included in this study. Of the participants, 69·5 % were female, and 30·5 % were male. Additionally, 79·7 % reported having no health issues, and 24·7 % indicated receiving nutrition education.

**Table 1.** Socio-demographic characteristics of participants

		*N*	%	Mean	ss	*t/F*	*P*
Sex	Female	335	69·5	64·83	13.807		
	Male	147	30·5	58·53	15.078	−4·484	<0·001***
Marital status	Single	275	57·1	61·83	14.68		
	Married	207	42·9	64·34	14.12	−1·888	.060
Education status	Primary education	41	8·5	65·27	12.34		
	High School	101	21·0	65·59	12.26	1·395	.243
	University	306	63·5	60·91	15.77		
	Postgraduate	34	7·1	62·96	14.49		
Income status	My income is lower than my expenses	104	21·6	61·63	13.82		
	My income is equal to my expenses	130	27·0	63·52	14.90		
	My income is higher than my expenses	248	51·5	63·13	14.55	.544	.581
Smoking status	I used to smoke, but I no longer do	79	16·4	60·53	12.37		
	I have never smoked	231	47·9	62·73	14.32	1·825	.162
	I smoke	172	35·7	64·25	15.48		
Health issues	Yes	98	20·3	61·83	12.80		
	No	384	79·7	63·19	14.88	−.830	.407
Previous nutrition education status	Yes	119	24·7	68·69	13.50		
	No	363	75·3	61·02	14.30	5·145	<0·001***
	**Total**	**482**	**100·0**				

**Figure 2.**
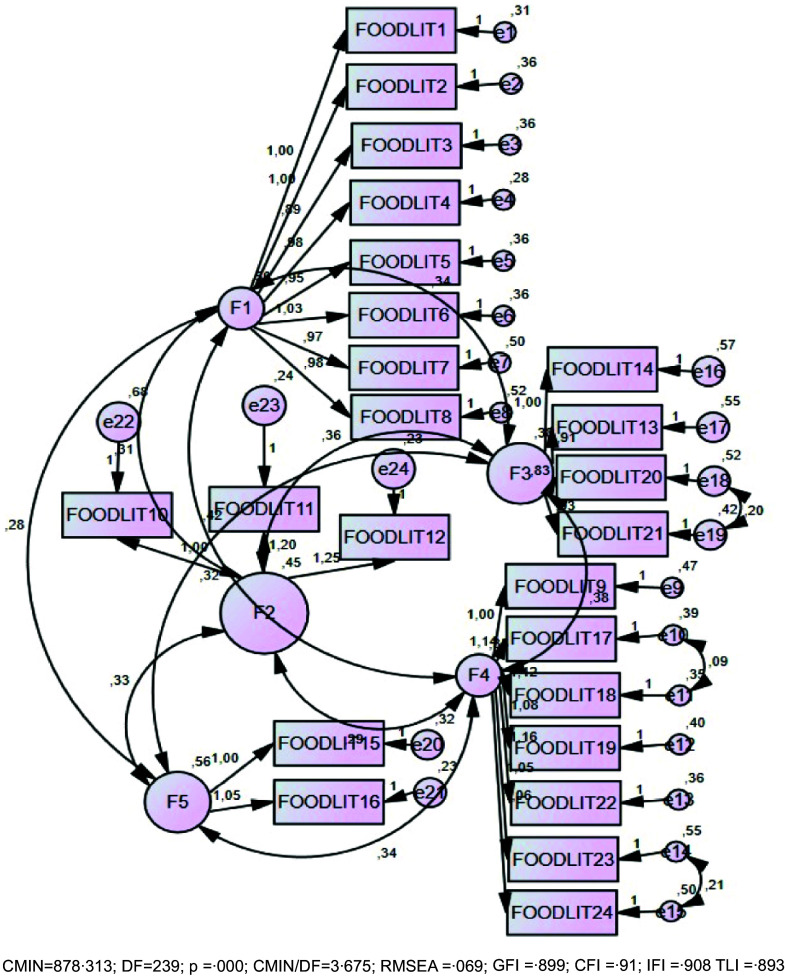
Figure 2 long description. Confirmatory factor analysis (CFA) results for the FOODLIT-TR-Tool.

In Table 2, the CFA measurement values for the variables in the FOODLIT-TR scale indicate that acceptable goodness-of-fit values were achieved for this sample (*χ* ^2^/df = 3·67, GFI = 0·90, Adjusted Goodness of Fit Index (AGFI) = 0·85, CFI = 0·91, Normed Fit Index (NFI) = 0·90, RMSEA = 0·07 and Standardized Root Mean Square Residual (SRMR) = 0·05). These CFA results demonstrate that the proposed model fits well with the data (Fig. 2).

**Table 2.** CFA goodness-of-fit index for the measurement model

	*χ* ^2^/sd	GFI	AGFI	CFI	NFI	RMSEA	SRMR
Normal value	<2	>0·95	>0·90	>0·95	>0·95	<0·05	<0·05
Acceptable value	<5	>0·90	>0·85	>0·90	>0·90	<0·10	<0·08
CFA measurement model	3·67	0·90	0·85	0·91	0·90	0·07	0·05

CFA, confirmatory factor analysis.

The Cronbach’s α internal consistency coefficient of the FOODLIT scale ranges between 0·66 and 0·91 across the sub-dimensions (Table 3). The Spearman–Brown coefficient ranges from 0·53 to 0·91, while the Guttman split-half coefficient varies between 0·60 and 0·91. These results indicate that the scale is ‘highly reliable’.

**Table 3.** Internal consistency and reliability analysis of sub-dimensions

Sub-dimension	Item	Item-total correlation	Cronbach’s alpha if item deleted	Cronbach’s alpha for sub-dimension	Spearman-brown reliability coefficient	Guttman
Culinary competencies	F1	0·755	0·892	0·908	0·907	0·907
	F2	0·729	0·894			
	F3	0·685	0·898			
	F4	0·757	0·892			
	F5	0·698	0·897			
	F6	0·727	0·894			
	F7	0·662	0·900			
	F8	0·635	0·903			
Production and quality	F10	0·546	0·864	0·810	0·843	0·765
	F11	0·718	0·682			
	F12	0·729	0·667			
Selection and planning	F9	0·534	0·873	0·875	0·784	0·776
	F17	0·685	0·854			
	F18	0·725	0·849			
	F19	0·658	0·857			
	F22	0·700	0·852			
	F23	0·634	0·861			
	F24	0·661	0·857			
Environmentally safe	F13	0·526	0·490	0·661	0·529	0·600
	F14	0·465	0·575			
	F20	0·428	0·620			
	F21	0·526	0·490			
Origin	F15	0·682	–	0·811	0·811	0·811
	F16	0·682	–			

Test-retest reliability was examined to assess the temporal stability of the FOODLIT-TR scale. The scale was re-administered to fifty-seven participants after a 30-day interval. Internal consistency across the two measurement occasions was high, with a Cronbach’s α coefficient of *α* = 0·957.

Temporal stability was further evaluated using the intraclass correlation coefficient based on a two-way mixed-effects model, in which subjects were treated as random effects and measurement occasions as fixed effects. The intraclass correlation coefficient for single measurements was 0·917 (95 % CI: 0·856–0·953) and for average measurements was 0·957 (95 % CI: 0·922–0·976), indicating excellent reliability over time.

In addition, the F-test was statistically significant, F(45, 45) = 23·239, *p* < 0·001, confirming that the observed consistency between measurements was not due to random variation.

**Figure 3.**
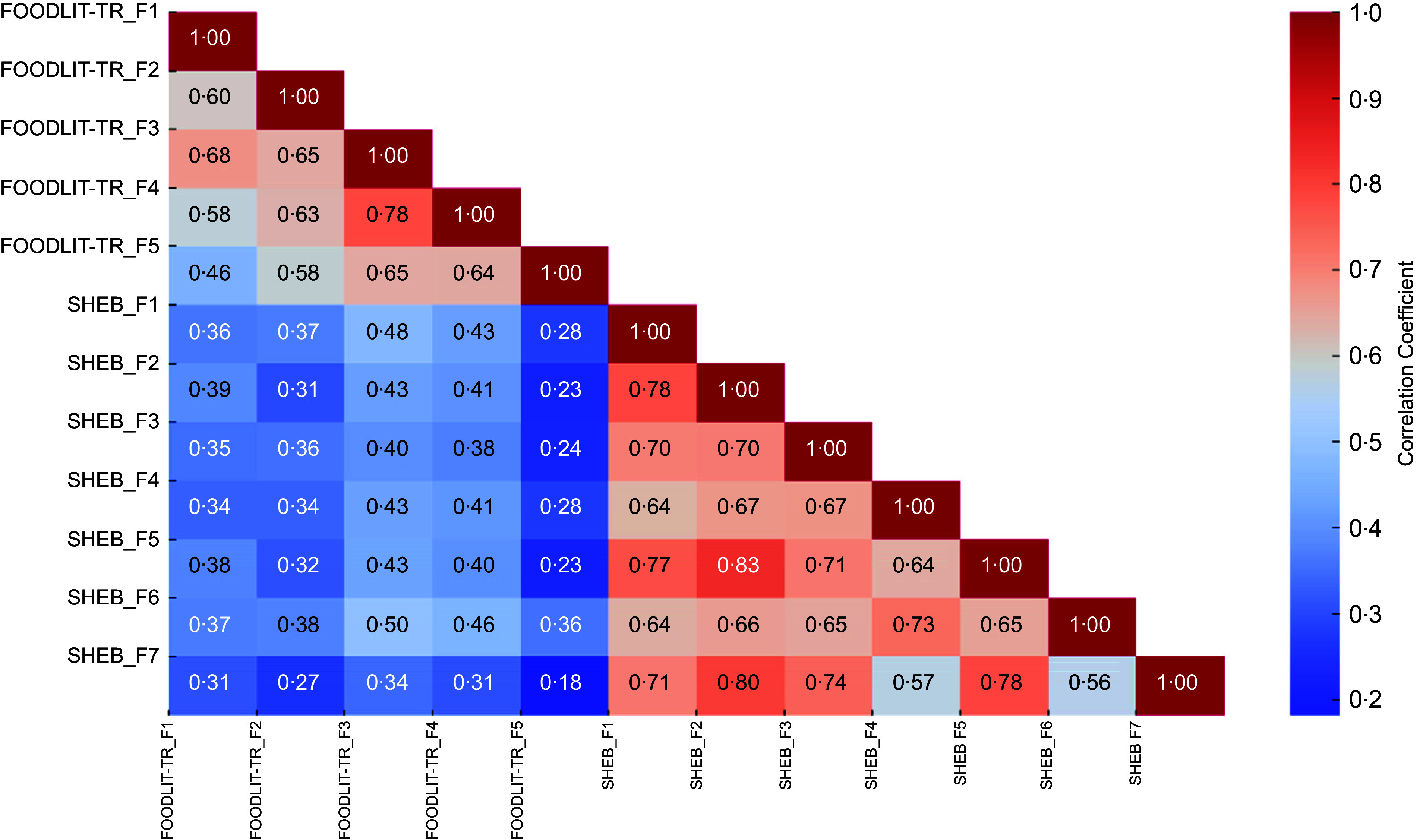
Correlation matrix of FOODLIT-TR and SHEB dimensions. FOODLIT-TR_F1: ‘Culinary Competencies’; FOODLIT-TR_F2: ‘Production And Quality’; FOODLIT-TR_F3: ‘Selection And Planning’; FOODLIT-TR_F4: ‘Environmentally Safe’; FOODLIT-TR_F5: ‘Origin’; SHEB_F1: ‘Quality Labels (Regional And Organic)’; SHEB_F2: ‘Seasonal Food And Avoiding Food Waste’; SHEB_F3: ‘Animal Welfare’; SHEB_F4: ‘Meat Reduction’; SHEB_F5: ‘Healthy And Balanced Diet’; SHEB_F6: ‘Local Food’; SHEB_F7: ‘Low Fat’.

According to the correlation analysis displayed in Fig.3, significant positive relationships were identified between the factors of the FOODLIT and SHEB scales. Specifically, medium-level correlations were found between FOODLIT_F1 (Culinary Competencies) and SHEB_F1 (Quality Labels, Regional and Organic) (*r* = 0·361) as well as SHEB_F2 (Seasonal Food and Avoiding Food Waste) (*r* = 0·388). Additionally, positive relationships were observed between FOODLIT_F1 and SHEB_F4 (Meat Reduction) (*r* = 0·337), SHEB_F5 (Healthy and Balanced Diet) (*r* = 0·377), SHEB_F6 (Local Food) (*r* = 0·372) and SHEB_F7 (Low Fat) (*r* = 0·314) (*p* < 0·05). It was also found that the FOODLIT_F2 (Production and Quality) factor positively correlated with SHEB_F1 (Quality Labels, Regional and Organic) (*r* = 0·373) and SHEB_F2 (Seasonal Food and Avoiding Food Waste) (*r* = 0·306).

In the sex-based comparison shown in Table 4, women scored significantly higher than men in the dimensions of culinary competencies (*p* < 0·001), production and quality (*p* = 0·009), choice and planning (*p* = 0·009) and environmental security (*p* = 0·002). However, no significant difference was found between sexes regarding awareness of food origin (*p* = 0·344).

**Table 4.** Comparison of FOODLIT sub-dimensions by sex

	Sex	*N*	Mean	SS	*t*	*p*
Culinary competencies	Male	147	21·02	6·096	−6525	<0·001***
	Female	335	24·60	5·276		
Production and quality	Male	147	7·74	2·468	−2608	0·009**
	Female	335	8·39	2·533		
Selection and planning	Male	147	17·91	5·152	−2620	0·009**
	Female	335	19·16	4·685		
Environmentally safe	Male	147	7·31	2·183	−3148	0·002**
	Female	335	7·98	2·115		
Origin	Male	147	4·52	1·618	−0,947	0·344
	Female	335	4·68	1·742		

***p < 0·001, **p < 0·01, * *p* < 0·05.

## Discussion

Nutrition literacy encompasses individuals’ abilities to make healthy, sustainable and ethical food choices and to implement these choices consciously^(24)^. It extends beyond personal health maintenance, including a strong relationship with environmental sustainability and ethical consumption behaviours. While nutrition literacy guides individuals in making healthy and balanced dietary choices, sustainable nutrition broadens this perspective by considering these choices’ environmental, economic and societal impacts. The increasing global environmental challenges and limited natural resources underscore the importance of promoting sustainable nutrition practices. In this context, sustainable nutrition literacy holds significant potential for fostering healthy individuals and ensuring environmental sustainability. This study aims to evaluate the psychometric properties of the FOODLIT scale, developed as a tool for measuring sustainable nutrition literacy.

Although a Turkish validity and reliability study for the FOODLIT-Tool was recently published^(25)^, test-retest reliability was not conducted in that study. While the linguistic adaptation and content validity of the scale were carried out, the absence of a temporal stability assessment limits the comprehensive evaluation of the scale as a reliable and valid measurement tool in Turkish culture. This study’s translation and back-translation process ensured language validity and strengthened semantic coherence. Additionally, with contributions from experts in nutrition and dietetics, content validity was evaluated, and the scale’s content alignment was thoroughly analysed. With a 30-d interval, the test-retest method was used to assess the scale’s reliability over time. These additional analyses comprehensively evaluated the FOODLIT-TR scale’s validity and reliability, demonstrating its suitability as a robust measurement tool for Turkish culture.

To evaluate the psychometric properties of the FOODLIT-TR scale, CFA was used to test the factor structure, and internal consistency and construct validity analyses were conducted to assess its reliability and validity^(26,27)^. The fit indices were *χ* ^2^/df=3·67, GFI = 0·90, AGFI = 0·85, CFI = 0·91, NFI = 0·90, RMSEA = 0·07, SRMR = 0·05, aligning with the accepted threshold values: *χ* ^2^/df < 5·0, CFI, GFI, TLI, NNFI, and IFI ≥ 0·90, AGFI > 0·85, RMSEA and SRMR ≤ 0·10. These CFA results indicate that the proposed model fits the data at an acceptable level. According to widely accepted criteria in the literature, CFI and NFI values above 0·90 indicate good model fit^(28)^. Additionally, the RMSEA and SRMR values within acceptable limits show that the model maintains low measurement error and supports the scale’s construct validity. The CFA results demonstrate that the proposed model provides sufficient fit with the data, confirming the scale’s factor structure reliably.

It is important to note that the *χ* ^2^ statistic is highly sensitive to sample size, especially in studies with larger samples such as this one (*N* 482). Even minor discrepancies in the model can result in a higher *χ* ^2^ value, which in turn inflates the *χ* ^2^/df ratio^(27,29)^. While a *χ* ^2^/df ratio below 3 is often considered ideal, values up to 5 are acceptable depending on the context and sample size^(30,31)^. In this study, the *χ* ^2^/df ratio of 3·67 is within this acceptable range and is supported by other fit indices that meet established benchmarks (e.g. CFI and TLI > 0·90; RMSEA < 0·08), confirming the robustness of the model^(28)^.

In the previous validity and reliability study, the fit indices obtained (*χ* ^2^/df=1·257, CFI = 0·991, GFI = 0·977, TLI = 0·990, NNFI = 0·990, IFI = 0·991, AGFI = 0·971, SRMR = 0·061 and RMSEA = 0·028) indicated robust model fit with the data^(25)^. The fit indices from the original study by Rosas et al. (*χ* ^2^/df = 3·958, SRMR = 0·055, RMSEA = 0·055, CFI = 0·907 and GFI = 0·917) were comparatively lower^(11)^. These findings are consistent with structural validity results in similar scale development studies in the literature^(27)^, supporting using the FOODLIT-TR scale as a reliable measurement tool.

To assess internal consistency, Cronbach’s α values were calculated for the sub-dimensions: culinary competencies, production and quality, selection and planning, environmentally safe and origin, with scores of 0·907, 0·765, 0·776, 0·600 and 0·811, respectively, demonstrating high reliability overall. Cronbach’s α values above 0·70 indicate high reliability for the scale^(32)^. In this context, the FOODLIT-TR scale shows satisfactory internal consistency across its sub-dimensions. The highest internal consistency value in the culinary competency dimension (*α* = 0·91) strongly supports the accuracy of the items within this dimension. The high Spearman-Brown and Guttman split-half coefficients (ranging from 0·53 to 0·91) further reinforce the scale’s consistency. Compared with previous studies, the reliability coefficients for the production, quality and environmentally safe sub-dimensions appear improved in the Turkish adaptation. In the adaptation study by Ertaş Öztürk et al., Cronbach’s α values were 0·871, 0·883, 0·903, 0·763 and 0·866, respectively^(25)^, while the original scale values were reported as 0·89, 0·65, 0·87, 0·76 and 0·87 for the exact dimensions^(11)^. These findings indicate that the FOODLIT-TR successfully adapts as a valid and reliable measurement tool for Turkish culture and enhances the reliability of specific dimensions from the original scale. Our findings align with the literature highlighting the importance of reliability in scale development studies^(26)^, supporting the high internal consistency of the Turkish version.

When examining the relationship between the FOODLIT-TR Scale and the Sustainable and Healthy Eating Behaviors Scale, a significant positive correlation was found between the two. The FOODLIT-TR scale measures individuals’ culinary competencies and food literacy levels, while the Sustainable and Healthy Eating Behaviors Scale evaluates participants’ sustainability-focused dietary choices. The positive correlation (e.g. *r* = 0·65) suggests that individuals with higher culinary skills and food literacy also tend to make more sustainable and healthy dietary choices. Literature also supports a relationship between these two concepts; individuals with high food literacy are more capable of developing sustainable eating habits^(24,33)^. This underscores that sustainable nutrition contributes to both environmental and individual health.

Correlation analysis revealed positive relationships among the sub-dimensions of these scales. Notably, there are strong relationships between culinary competencies and selection and planning (*r* = 0·680) and environmental security (*r* = 0·579), indicating that the sub-dimensions form a consistent structure, effectively representing each intended concept. The positive correlations support the scale’s construct validity, which tests whether a scale accurately measures the intended concept^(20)^. These findings offer important insights into how culinary competence and awareness of production and quality impact individuals’ sustainable food choices, ethical responsibilities and healthy eating behaviours.

For instance, the moderate positive relationship between culinary competencies and quality labels (*r* = 0·361) suggests that as individuals’ culinary skills increase, so does their awareness of regional and organic products. This finding aligns with other studies indicating that individuals with high food literacy are more inclined towards sustainable eating habits^(24,34)^. Additionally, the positive relationship between culinary competence in seasonal food consumption and food waste prevention (*r* = 0·388) highlights the contribution of culinary skills to sustainability. The literature also frequently emphasises the link between seasonal eating and environmental impact reduction^(35)^.

This positive relationship between culinary competence and seasonal food consumption and food waste prevention suggests that more competent individuals in the kitchen are more aware of sustainability. This finding shows that sustainable nutrition is directly related to environmental issues and daily kitchen practices. Similarly, the relationships between animal welfare, meat reduction and culinary competence (*r* = 0·351 and *r* = 0·337) indicate that individuals are becoming more sensitive to ethical food production and animal rights. Particularly in recent years, reducing meat consumption has been emphasised as an essential strategy in sustainable nutrition^(36)^. The positive relationship between healthy and balanced eating and culinary competence (*r* = 0·377) shows that culinary skills contribute to individuals’ health-focused choices, supporting the impact of food literacy on healthy eating habits as documented in the literature^(24)^. This highlights that time spent in the kitchen and food knowledge increase awareness of environmental and ethical issues and health-related awareness. The positive relationship between local food preferences and culinary competence shows that competent individuals are more interested in local and sustainable foods. Given the importance of local food choices for sustainability, it can be said that competent individuals in the kitchen are more supportive of local production.

Findings related to the production and quality sub-dimension show individuals’ interest in sustainable production processes and their impact on sustainable food consumption habits. The positive relationship between regional and organic labels and awareness of production and quality (*r* = 0·373) suggests that individuals make more ethical and environmentally aware food choices. These results align with findings from the literature showing that individuals prioritising production quality tend to develop sustainable eating habits^(37)^.

The analysis by sex shows that women scored significantly higher than men in the sub-dimensions of culinary competence, production and quality, selection and planning and environmental security, which are central aspects of sustainable nutrition literacy. This finding aligns with general trends in the literature, suggesting that women are more conscious of sustainable and ethical nutrition^(38–41)^. Some studies have reported that women prefer low-carbon, local and organic products more than men and have more developed sustainability awareness^(42,43)^. Traditional social roles lead women to be more active in household food preparation, but studies going beyond these roles reveal that women develop a deeper awareness of environmental issues^(39,44–46)^. It is suggested that this trend arises from women’s tendency to consider long-term benefits, such as protecting the environment, considering the well-being of future generations, and safeguarding health^(47)^. Although no significant sex difference was found in awareness of food origin, differences in other dimensions show that women have a more vital awareness of sustainable nutrition, indicating that sex may be an essential determinant in nutrition literacy.

The findings of this study confirm that the adapted FOODLIT-TR tool effectively captures the multidimensional aspects of food literacy, particularly in the context of sustainability. One notable observation is the higher scores among women in culinary competence, production and quality, choice and planning and environmental security dimensions. This result aligns with existing literature, which suggests that women often engage more actively in household food practices and exhibit greater environmental awareness. However, beyond traditional roles, this could also reflect a growing societal emphasis on gendered responsibilities in sustainable practices. The positive correlations between FOODLIT-TR sub-dimensions and sustainable eating behaviours highlight the importance of integrating food literacy into public health strategies. For instance, the strong link between culinary competence and sustainable eating habits underscores the potential of educational interventions targeting practical food skills to influence dietary behaviours positively.

This study demonstrates the potential of the FOODLIT-TR tool to shape public health policies and educational programmes aimed at promoting sustainable nutrition in Turkey. By providing a reliable measure of sustainable nutrition literacy, the tool can help policymakers identify gaps in food literacy and design targeted interventions to address these needs. For example, the tool’s ability to measure culinary competencies and sustainability-related behaviours makes it particularly useful for developing campaigns to reduce food waste, encourage plant-based diets and promote local food systems. Additionally, the FOODLIT-TR tool can support educational initiatives by assessing the impact of nutrition education programmes on participants’ knowledge and behaviours. Tailored curricula for schools, universities and community education programmes could incorporate the tool to measure progress and refine strategies.

The slight deviations in model fit indices, such as the *χ* ^2^/df ratio exceeding the ideal threshold, are reflective of the challenges in modeling complex, multidimensional constructs in large samples. Nevertheless, the strong performance of other fit indices validates the robustness of the adapted tool. These findings suggest that sustainable food literacy is not only a personal attribute but also shaped by cultural, environmental, and educational factors. Future interventions should therefore consider these dimensions holistically to maximize their impact on promoting sustainable food systems.

The findings from this study offer valuable insights for advancing global sustainability within food systems. The FOODLIT-Tool enables policymakers to identify gaps in food literacy and design targeted policies that promote sustainable food practices and improve dietary habits. The results also guide the development of educational programmes, such as those aimed at reducing food waste, encouraging sustainable diets and enhancing food preparation skills, tailored to the needs highlighted by the tool.

Additionally, the FOODLIT-Tool demonstrates a high level of discriminatory power, as evidenced by the results of the independent samples t-test presented in supplementary. Accordingly, food literacy levels can be categorised as follows: < 50 or 55 points: Low food literacy, 50–75 points: Moderate food literacy, >75 points: High food literacy. These categorisations provide a clear framework for assessing food literacy levels and offer practical guidance for tailoring interventions and programmes to address specific needs. Finally, the FOODLIT-Tool provides a foundation for future research, enabling longitudinal studies and cross-cultural applications to explore food literacy’s role in sustainability. This adaptable framework positions the tool as a key resource for fostering informed and sustainable decision-making within food systems.

As in many cross-cultural adaptation studies, the assessment of language validity in the present study relied on correlational evidence. While this approach provides useful preliminary information regarding the consistency between the original and translated versions of the scale, it does not fully capture measurement equivalence, which should be addressed in future studies using agreement-based methods.

Several limitations of this study should be acknowledged when interpreting the findings. First, the use of snowball sampling may have introduced selection bias, resulting in a sample that is not fully representative of the general Turkish adult population. This sampling approach may have contributed to a more homogeneous participant profile, which in turn could have influenced the observed factor structure and inflated internal consistency coefficients, such as Cronbach’s α. Therefore, the psychometric properties reported in this study should be interpreted as sample-specific rather than universally generalisable.

Second, data were collected using self-reported measures, which are inherently susceptible to social desirability and response biases. Participants may have overreported socially desirable behaviours related to sustainable and healthy eating, potentially leading to overestimated associations between food literacy and sustainability-related behaviours.

In addition, both exploratory and confirmatory factor analyses were conducted on the same sample, which may increase the risk of model overfitting. Consequently, the confirmatory findings should be interpreted as preliminary evidence supporting the proposed factor structure rather than definitive model validation. Although the factor structure was examined using sequential EFA and CFA within the same dataset, independent cross-validation was not performed. While this approach is considered acceptable in applied scale adaptation research when findings are interpreted cautiously, the confirmatory results should be regarded as preliminary structural evidence. Future studies employing independent samples are recommended to evaluate further the stability and generalisability of the FOODLIT-TR factor structure.

Finally, the study sample was limited to adults aged 18–65 years residing in Turkey, restricting the generalisability of the findings to younger, older or culturally distinct populations. Future studies employing probabilistic sampling methods, objective behavioural measures and more diverse age groups are warranted to further validate the FOODLIT-TR scale.

## Conclusions

The findings of this study indicate that culinary competence and awareness of food production and quality play important roles in shaping individuals’ food choices with respect to sustainability, ethics and health. Individuals with higher levels of food literacy appear more likely to make conscious dietary choices that align with environmental sustainability and ethical considerations.

Furthermore, the validity and reliability analyses suggest that the FOODLIT-TR scale demonstrates satisfactory psychometric properties and constitutes a comprehensive and reliable instrument for assessing sustainable nutrition literacy among Turkish adults. In this respect, the present study contributes to the growing body of literature on food literacy and sustainability by providing a culturally adapted and psychometrically evaluated measurement tool.

However, these findings should be interpreted considering the study’s limitations, including its cross-sectional design, non-probabilistic sampling strategy and reliance on self-reported data. Within these limitations, the FOODLIT-TR scale may serve as a useful assessment tool for future research, and intervention studies aimed at understanding and improving sustainable eating behaviours. Further studies employing representative samples, longitudinal designs and objective measures are needed before drawing conclusions regarding its applicability in large-scale public health initiatives or policy-making contexts.

## Figure 1. Long description

The flowchart outlines the study design for evaluating sustainable nutrition among adults aged 18-65 in Turkey. The study population includes adults aged 18-65, with 482 participants achieved. The Google Forms survey was shared via email and WhatsApp using snowball sampling. Eligibility criteria include adults aged 18+ residing in Turkey, fluent in Turkish, with voluntary participation and no health issues preventing survey completion. Instrument adaptation involved obtaining permission for the FOODLIT-Tool adaptation, completing a six-stage translation process following Beaton et al. (2000) guidelines, and conducting a pre-test with a similar sample group. Reliability and validity were assessed through content validity evaluation by 7 nutrition experts, calculating S-CVI and I-CVI, and conducting test-retest reliability with 57 volunteers over a 30-day interval. Data collection instruments include the FOODLIT-Tool and the Sustainable Healthy Eating Behavior Scale, with the collection period set for July-August 2024. Statistical analysis involves descriptive statistics, validity testing, and reliability testing.

Navigate back to Figure 1..

## Figure 2. Long description

A diagram representing the confirmatory factor analysis results for the FOODLIT-TR-Tool. The diagram includes several factors (F1, F2, F3, F4, F5) and their relationships with various FOODLIT items. Each factor is connected to specific FOODLIT items through arrows indicating the direction and strength of the relationships. The factors are interconnected, showing the overall structure and flow of the analysis. The diagram also includes error terms (e1, e2, etc.) associated with each FOODLIT item, indicating the variance not explained by the factors. The confirmatory factor analysis results are summarized with statistical values such as CMIN, DF, p-value, CMIN/DF, RMSEA, GFI, CFI, IFI, and TLI.

Navigate back to Figure 2..
